# Recurrent ligneous conjunctivitis after cataract surgery in a 67-year-old male patient: a case report

**DOI:** 10.1186/s12886-022-02329-7

**Published:** 2022-03-05

**Authors:** Amine Maamri, Elena Zemova, Kayed Moslemani, Fidelis Flockerzi, Berthold Seitz

**Affiliations:** 1 Department of Ophthalmology, Saarland University Medical Center, Kirrbergerstraße 100, Bld. 22, 66421 Homburg/Saar, Germany; 2 Department of General and Special Pathology, Saarland University Medical Center, Homburg/Saar, Germany

**Keywords:** Ligneous conjunctivitis, Granuloma, Plasminogen, Cataract surgery

## Abstract

**Background:**

Ligneous conjunctivitis is a rare form of chronic pseudomembranous conjunctivitis which usually starts during infancy. We report on an unsual case of recurrent ligneous conjunctivitis after cataract surgery in a 67-year-old male patient.

**Methods:**

The equipment used for the slit-lamp images was a Haag Streit slit lamp BX900 Sn 00,406 with 16 × magnifications. The used batch number of the camera was sn00406 and the software was from the company CCS Pawlowski Merge Eye. There were no filters used. The images were saved with a resolution of 300 DPI. Neither downstream nor averaging was used to enhance the resolution of the image in the case presentation section or the figure legend.

The equipment used for the cross-sectional histologic images was a Zeiss Axioskop 40 microscope with an objective lens Zeiss A-Plan × 20/0.45 (Zoom 6.3 × TV 2/3″″C). The used camera was AxioCam MRc5 and the software was ZEN 3.2. The cross-sectional histologic images were saved with a resolution of 2584 × 1936 Pixels. Neither downstream nor averaging was used to enhance the resolution of the image in the case presentation section or the figure legend.

**Case presentation:**

This is a rare case report of ligneous conjunctivitis in a 67-year-old male patient who presented a recurrent conjunctival granuloma after five excisions following cataract surgery in his left eye. We performed a tumor excision with free conjunctival autograft. The histology showed a fibrin crust including macrophages, granulocytes, lymphocytes, and reactively altered squamous cell nests. These findings were consistent with a ″pseudomembrane in conjunctivitis lignosa″. We administered a topical combination of plasminactivator, heparin, cortisone and cyclosporine.

**Conclusion:**

This treatment with the combination of plasminactivator, heparin, cortisone and cyclosporine has proven to be effective in preventing the recurrence of ligneous conjunctivitis.

## Background

Ligneous conjunctivitis (LC) is a rare form of chronic conjunctivitis [1]. It usually occurs during infancy and childhood with a slight predominance in females whereas less than 20% occur in the fourth and fifth decade of life [1, 2]. The first report of ligneous conjunctivitis in a 46-year-old man with bilateral pseudomembraneous conjunctivitis was published by Bouisson as early as 1847 [3]. Overall, less than 200 cases have been reported in the literature up until 2021 [1, 4].

## Case presentation

A 67-year-old man presented at our Department of Ophthalmology for treatment recommendation of recurrent conjunctival ″granuloma″ in his left eye. For the first time, the conjunctival granuloma occurred within the first six weeks after cataract surgery. One excision of such a lesion in the right eye and 5 excisions in the left eye had already preceded. The last surgical treatment in the left eye was performed only 4 weeks earlier.

This time, the patient complained of decreased visual acuity, foreign body sensation, and an increasingly hanging left eye lid.

Apart from the aforementioned operations, the patient had no other ophthalmological problems. Systemically the patient had arterial hypertension and hypothyroidism.

**Fig. 1:**
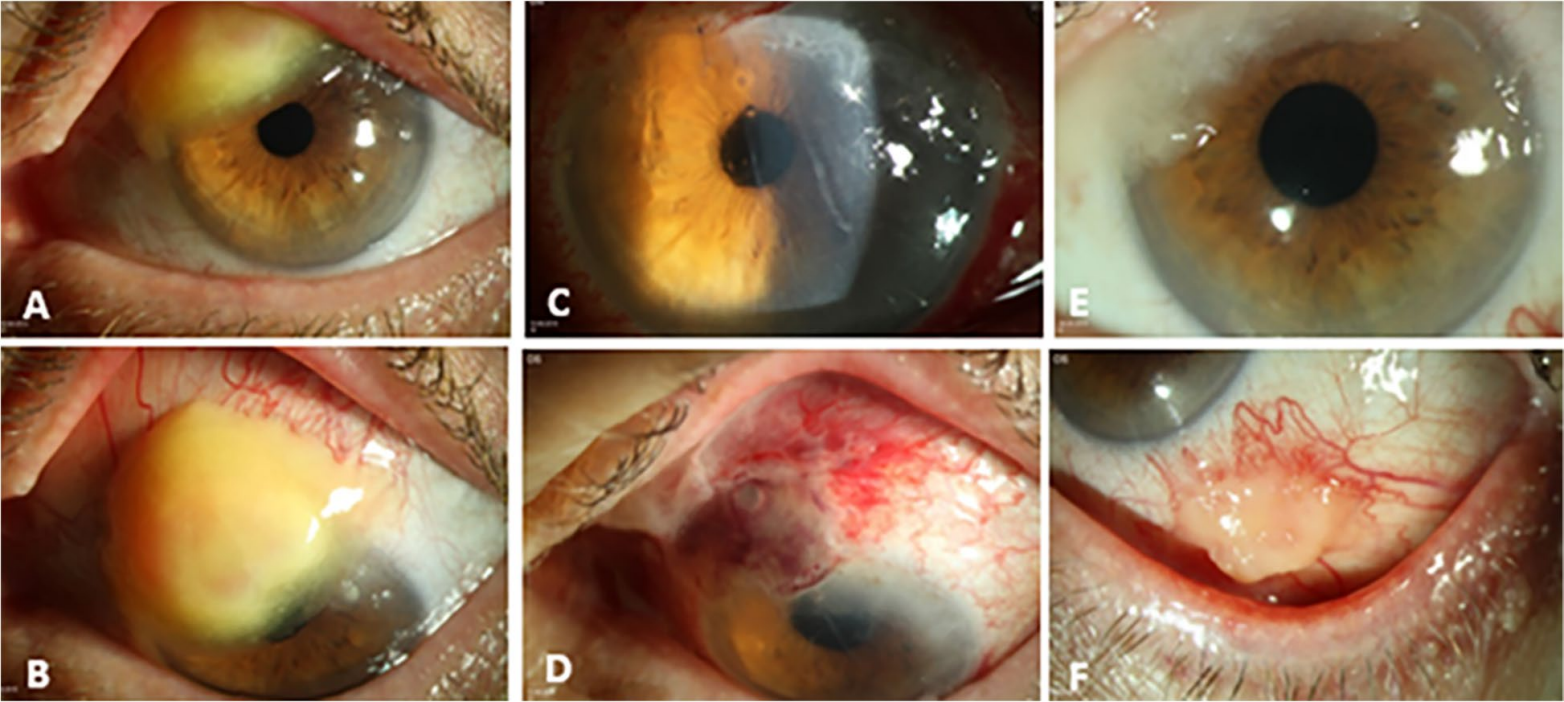
Slit-lamp images of the left eye: preoperative slit-lamp image (**A**, **B**) showing a wood-like granulation tissue extending from 10 over 12 to 1 o'clock from the limbus to the midperipheral cornea. The first postoperative day showed a well localized autograft after complete removal of the tumor and autologous conjunctival transplantation (**C**, **D**). Two weeks postoperative slit-lamp image (**E**, **F**) showing a recurrence of the conjunctival lesion at the original excision site from 10 to 1 o´clock and a second new lesion at 5 o´clock at the excision site of the conjunctival autograft

At the time of the initial presentation, the best corrected visual acuity (BCVA) was 20/40 on the left. The left eye presented a hanging eye lid to the middle of the pupil and a wood-like granulation tissue of the bulbar conjunctiva extending from 10 over 12 to 1 o'clock from the limbus to the midperipheral cornea (Fig. 1A and B). Otherwise, there was a regular pseudophakia. In the right eye, BCVA was 20/20 and there was no recurrence of the reported “granuloma”. Intraocular pressure was 14 mmHg in the right eye and 15 mmHg in the left eye. The funduscopic results showed no abnormalities bilaterally.

**Fig. 2:**
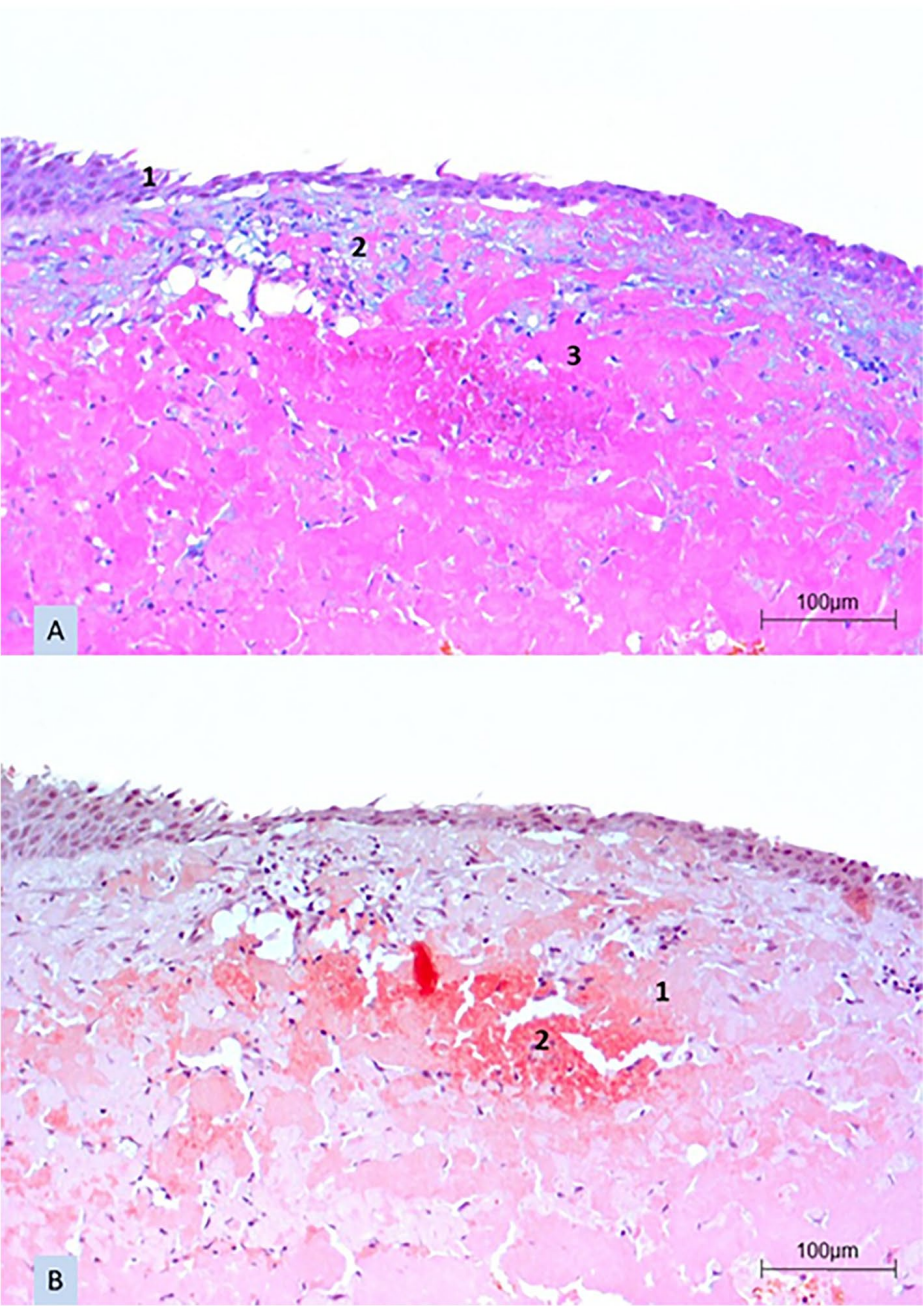
A cross-sectional histologic specimen of the conjunctiva after tumor excision in the left eye. The Alcian blue staining (**A**) showed an intact epithelium (**A1**) and a subepithelial accentuated chronic inflammation with deposits of mucin (**A2**) and eosinophilic hyaline material (**A3**). In the Congo-red staining (**B**), the cross section of the conjunctiva showed a negative staining, no apple green birefringence in polarized light (**B1**) and only red-stained erythrocytes (**B2**)

A tumor excision with free conjunctival autograft was performed in the left eye. The first postoperative day revealed a well localized autograft. The granuloma was completely removed (Fig. 1C and D). After excision in our clinic, the patient was treated with a topical therapy consisting of prednisolone acetate 3 times a day, fluoroquinolone 3 times a day and lubricant 3 times a day. The patient was discharged after 2 nights with a well-adapted conjunctival graft. Later, histology showed an intact epithelium and a subepithelial accentuated chronic inflammation with deposits of mucin and eosinophilic hyaline material in the Alcian blue staining. In the Congo-red staining, the cross section of the conjunctiva showed a negative staining, no apple green birefringence in polarized light and only red-stained erythrocytes (Fig. 2A and B). These findings were consistent with a ″pseudomembrane in conjunctivitis lignosa″.

After two weeks, the patient presented with worse findings in the operated left eye. Clinical examination revealed two lesions: One at the original excision site and a second, new lesion at the excision site of the conjunctival graft at 5 o´clock (Fig. 1E and F).

Based on the histologic findings and literature research, we performed a blood coagulation analysis. This showed a plasminogen concentration of 1.5 g/l (normal value: 6-25 g/l) and a plasminogen activity of 21.98% (normal value: 75–150%) which confirmed the histological diagnosis of LC.

**Fig. 3:**
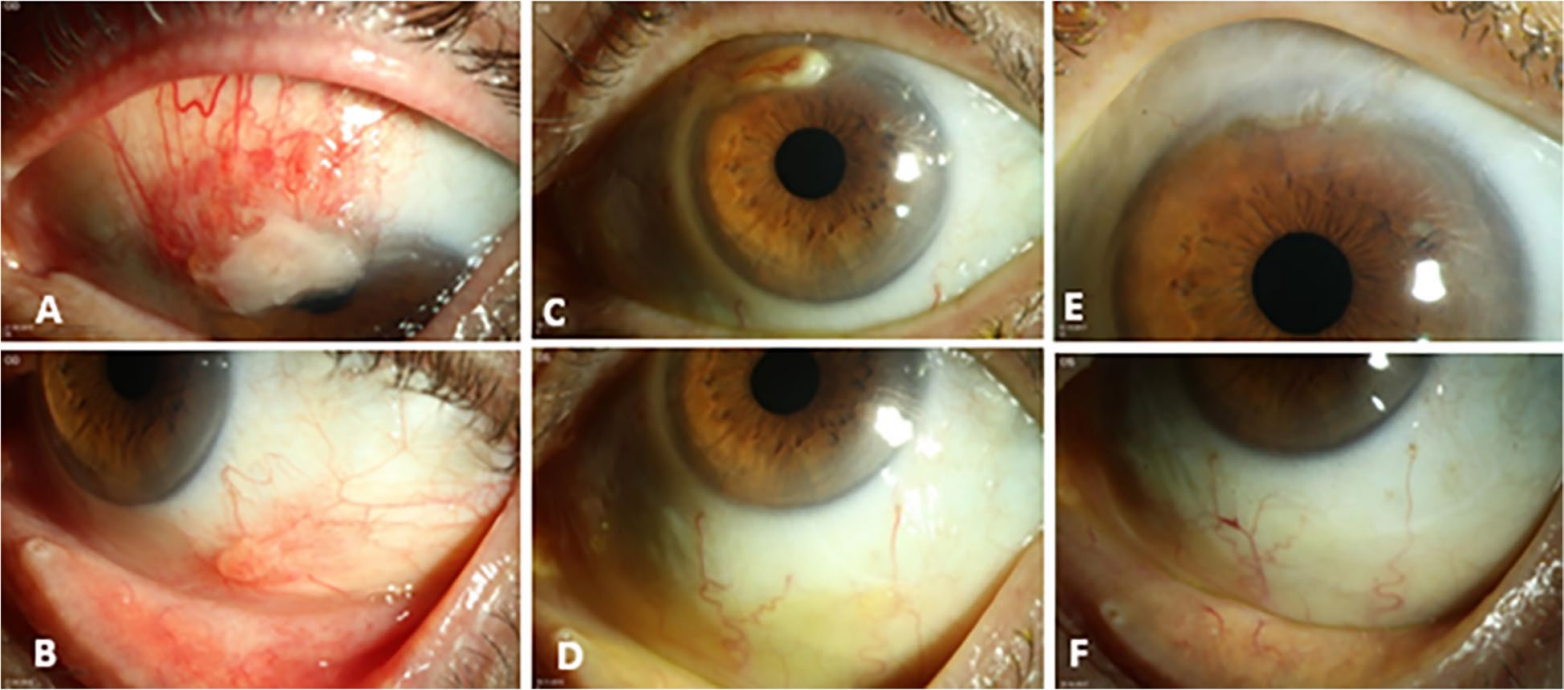
Slit-lamp images of the left eye: one month postoperative photographs (**A**, **B**) showing an improvement of the finding during therapy with alteplase. Three months postoperative slit-lamp photographs (**C**, **D**) showing a complete regression of the lesion at 5 o'clock and a minor residual lesion at 12 o'clock. Two years after the excision of the granulation tissue (**E**, **F**): flat conjunctival scar and avascular corneal scar from 10 to 1 o'clock at the limbus without any sign of recurrence

We started topical therapy with alteplase (plasminactivator) on an hourly basis, prednisolone acetate 3 times a day, cyclosporine 0,1% at night, and heparin 8 times a day. The patient did not receive a systemic therapy. We scheduled follow-ups at 1, 3, 5, 8, and 12 months after initiation of the therapy. At the first presentation after one month of topical therapy, we already saw a significant improvement of the findings (Fig. 3A and B). We continued with this therapy and planned a new check-up three months later. At that time, there was complete regression of the lesion at 5 o'clock and a minor residual lesion at 12 o'clock (Fig. 3C and D). Therefore, we decided to perform another excision while continuing the existing local therapy.

The postoperative follow-up after two months showed a vascularized arcuate corneal scar without signs of recurrence and the BCVA increased to 20/25 in the left eye so that topical therapy with alteplase eye drops could be reduced from hourly to five times daily. After eight months, the treatment with alteplase eye drops and heparin eye drops was reduced to two times daily for another three months. Even after five years without therapy, the findings were free of recurrence. The BCVA at that time was 20/20 in both eyes. Slit-lamp biomicroscopy showed an anterior segment within normal limits in the right eye. The left eye showed a flat conjunctival scar from 10 to 1 o'clock at the limbus without signs of recurrence (Fig. 3E and F).

## Discussion and conclusions

Ligneous conjunctivitis (LC) is characterized by the development of firm fibrin-rich, wood-like pseudomembraneous prominent lesions. It usually involves the tarsal conjunctiva of the upper eyelid. Other sites of manifestation include pharynx, ears, gastrointestinal tract, respiratory tract, and female genitourinary tract [5]. Our patient had no extraocular manifestations.

The disease may be inherited in an autosomal recessive manner [4]. However, in predisposed patients it may also be triggered sporadically, by local inflammation, microtrauma, or surgical procedures of the eyes [5]. In our case, an uncomplicated cataract surgery was the presumed triggering factor for the onset of LC.

It has been shown that plasminogen deficiency (type 1) and dysplasminogenemia (type 2) play a major role in the etiology [6]. Plasminogen is a precursor protein synthesized by the liver. Plasminogen activators (e.g. alteplase used by our patient) cause plasminogen to be activated and form plasmin. The main function of plasmin is to cleave fibrin and thus inhibit fibrin cross-linking. In the case of plasminogen deficiency, fibrin cannot be dissipated and, therefore, wound healing stops at the granulation stage. Greasy deposits typically appear on the surface of the wound.

The diagnosis of LC is made based on clinical findings, plasminogen activity and/or plasminogen antigen serum levels, plasminogen gene analysis, and typical histopathological findings [5–9].

Our patient showed a deficiency of plasminogen and plasminogen activity.

The histologic examination of pseudomembranes from affected eyes typically shows a thinned epithelium with subepithelial deposits of amorphous hyalin-like eosinophilic material and foci of persisting granulation tissue with inflammatory cells such as lymphocytes, plasma cells, and granulocytes.

Plasmin plays an important role in wound healing because it contributes to the migration of fibrin-rich extracellular matrix in healing skin. Furthermore, plasmin facilitates keratinocyte division, migration, and differentiation, thus supporting closure of skin wounds [5].

Previous treatment options included corticosteroids, topical hyaluronidase, alpha-

chymotrypsin, topical heparin, oral contraceptive, and immunosuppressive agents (such as azathioprine and cyclosporine A) [5, 6, 10]. After discovering that the etiology of conjunctivitis lignosa is plasminogen deficiency, specific treatment options such as fresh frozen plasma (FFP), plasmin, and plasminogen were added [7, 9, 11–16]. Although treatment with FFP and plasminogen results in regression of the ligneous membranes, the effects of additional immune suppressive agents may be used as an adjuvant treatment to suppress concomitant inflammation. Specific therapy with glu– or lys–plasminogen concentrates is still under investigation [11, 15, 16].

There are 3 principles of the therapy: fresh Frozen Plasma (FFP) contains the missing plasminogen, heparin inhibits fibrin formation and ciclosporin reduces the local inflammation [17].

In our case, we initially failed to make the correct diagnosis and adequate treatment with the consequence of recurrence after the excision and conjunctival autograft. After the current histological diagnosis, we used a combination of alteplase, cortisone, cyclosporine, and heparin eye drops. Since FFP could not be prepared by the pharmacy at our hospital, we did not use it. However, our combination proved to be effective in treating LC as well as preventing recurrence. Since multiple relapses had been involved, we made the decision to treat our patient with the maximal reasonable therapy combining steroids and cyclosporin A. We consider that this is why the removal of the residual granuloma in our patient five months after many surgeries under appropiate therapeutic cover was not complicated by a recurrence.

We must admit that the good compliance of our patient played an important role in the success of the treatment. This required daily preparation of the eye drops with alteplase and their storage in the refrigerator. During the period of the patient's admission, this was achieved thanks to the good coordination between our ophthalmology department and the pharmacy at our hospital. Once the patient was discharged, it was essential to find a pharmacy that was able and ready to prepare the alteplase eye drops every day, as they were not freely available on the market.

For the preparation of the eye drops, Actilyse was dissolved and mixed as a dry substance within an appropriate solvent. Due to the enzyme activity, the solution can only be stored for about 24 h, so that the patient needed to receive freshly prepared eye drops from the pharmacy every day (including Sundays). This was successfully achieved not only thanks to the efforts of the doctors and pharmacists, but also thanks to the very good compliance and assiduity of the patient who kept collecting the eye drops from the pharmacy and instilling them at home throughout the entire treatment period of seven months.

In conclusion, ligneous conjunctivitis may occur on the bulbar conjunctiva in a 67-year-old male after uncomplicated cataract surgery. After haematological and histological confirmation of the diagnosis, a consequent topical therapy with alteplase, cortisone, cyclosporine, and heparin eye drops over 11 months was able to achieve regression of the process and avoid a recurrence for at least 5 years despite cessation of the treatment.

## Data Availability

The datasets used and/or analysed during the current case report are available from the corresponding author on reasonable request.

## Abbreviations

LC: Ligneous conjunctivitis
BVCA: Best visual corrected acuity
Ig: Immunoglobulins
FFP: Fresh frozen plasma
